# Treatment of pulmonary epithelioid hemangioendothelioma with combination chemotherapy: Report of three cases and review of the literature

**DOI:** 10.3892/ol.2013.1217

**Published:** 2013-02-28

**Authors:** BO YE, WANG LI, JIAN FENG, JIAN-XIN SHI, YONG CHEN, BAO-HUI HAN

**Affiliations:** 1 Department of Thoracic Surgery, Shanghai Chest Hospital, Shanghai Jiaotong University, Shanghai 200030;; 2 Renji-MedX Clinical Stem Cell Research Center, Renji Hospital, Shanghai Jiao Tong University School of Medicine, Shanghai 200127;; 3 Division of Medical Oncology, Shanghai Chest Hospital, Shanghai Jiaotong University, Shanghai 200030, P.R. China

**Keywords:** pulmonary epithelioid hemangioendothelioma, pulmonary tumors, chemotherapy, metastases

## Abstract

No standard therapy for pulmonary epithelioid hemangioendothelioma (PEH) has yet been established due to the rarity of the disease, the lack of clear standards for treatment and the partial-to-complete spontaneous regression. This report describes three cases of PHE manifested as bilateral intrapulmonary masses with an initial diagnosis conducted by thoracoscopic lung biopsy. These patients demonstrated a partial response to combination chemotherapy with carboplatin, paclitaxel, bevacizumab or endostar, and an improvement in clinical status. Furthermore, we reviewed the literature regarding such patients who received chemotherapy and immunotherapy; this indicated that patients with PEH demonstrated a good partial response to chemotherapy with carboplatin, paclitaxel, bevacizumab, thalidomide and α-interferon. Overall, combination chemotherapy regimens may hold therapeutic potential for the treatment of this rare disease.

## Introduction

Pulmonary epithelioid hemangioendothelioma (PEH) was originally named intravascular bronchiolo-alveolar tumor (IVBAT) by Dail and Liebow in 1975 (1). PHE is a rare vascular tumor of low-grade malignancy and there is no clear standard for treatment. PEH typically occurs as bilateral multiple nodules among young females. PEH rarely develops as a solitary lung nodule. Unilateral single nodules may be surgically removed. Patients with diffuse lung lesions are mainly treated with chemotherapy, although no single chemotherapy agent has demonstrated efficiency in treating PEH. We describe three patients with PEH who were treated with a combination of carboplatin, paclitaxel, bevacizumab or endostar. We also review the literature on such patients who received chemotherapy and immunotherapy. The study was approved by the Ethics Committee of Shanghai Chest Hospital, Shanghai Jiaotong University, Shanghai, China. Written informed consent was obtained from the patient.

## Case reports

### Case 1

**Figure 1:**
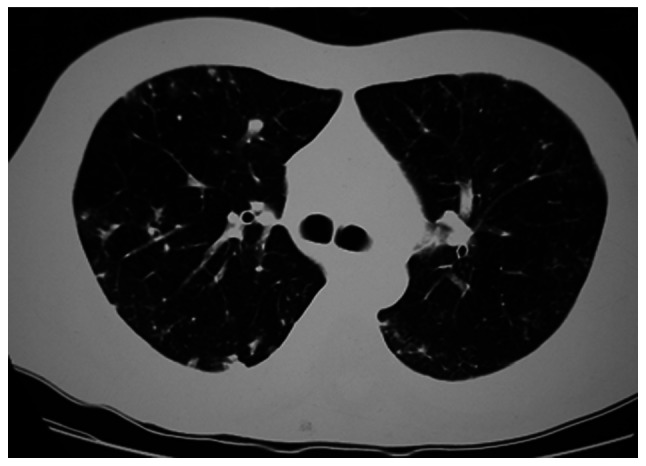
Thoracic computed tomography (CT) reveals multiple bilateral nodules of different sizes. Some of the nodules are surrounded by ground-glass opacities.

The patient was a 40-year-old Asian male with a four-month history of a dry cough, dyspnea and hemoptysis. The patient was a heavy smoker with an unremarkable medical history. A chest computed tomography (CT) scan revealed the presence of multiple nodules scattered in both lungs without hilar and mediastinal lymphadenopathy or pleural effusion (Fig. 1). Initially, a bronchofibroscope examination failed to reveal any abnormalities. In order to obtain a definitive diagnosis, the tissue specimens were taken by diagnostic right thoracoscopic lung biopsy.

**Figure 2:**
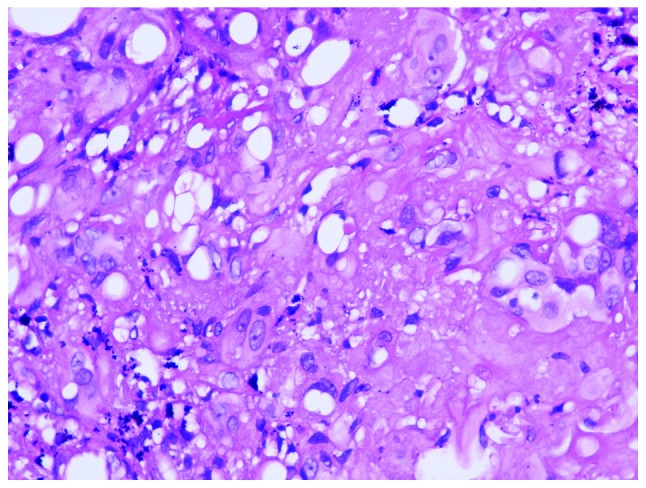
Several tumor cells show marked cytologic atypia with large hyper-chromatic nuclei, vesicular chromatin and prominent eosinophilic nucleoli (H&E staining; original magnification, ×400).

**Figure 3:**
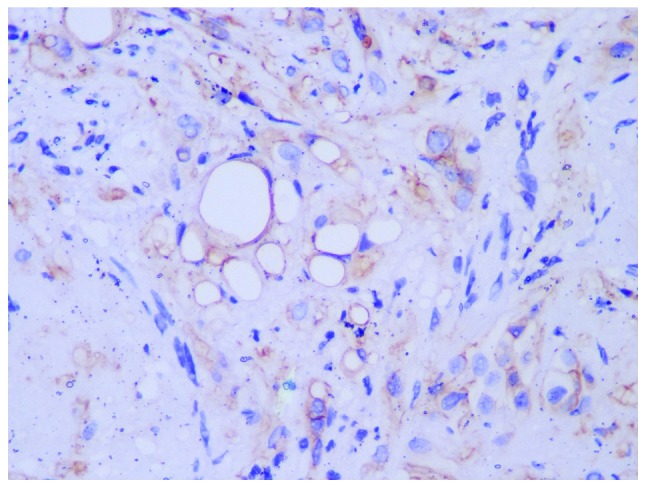
Immunostaining for CD34 reveals strong and diffuse positivity of epithelioid cells and prominent cytoplasmic vacuoles and lumens (original magnification, ×400).

The histological diagnosis of PEH was based on the pathological examination. The pathological examination of the biopsied specimen revealed that the center of the pulmonary nodule was sclerotic and hypocellular, with hyalinization and calcification. The tumor cells were round with abundant eosinophilic cytoplasm, intracytoplasmic vacuolization and a signet ring-like appearance (Fig. 2). Immunohistochemical analysis revealed that the tumor cells were positive for the endothelial markers, factor-VIII-related antigen and CD34 (Fig. 3).

**Figure 4:**
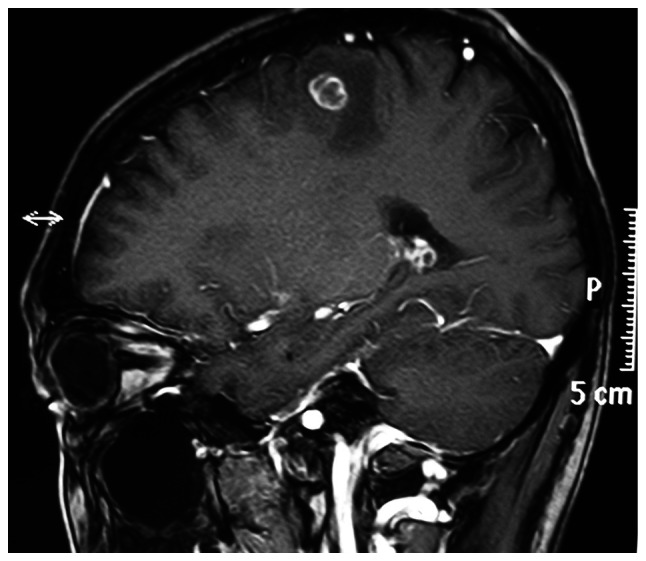
T1-weighted magnetic resonance imaging (MRI) section through the brain shows single brain metastasis from lung disease.

**Figure 5:**
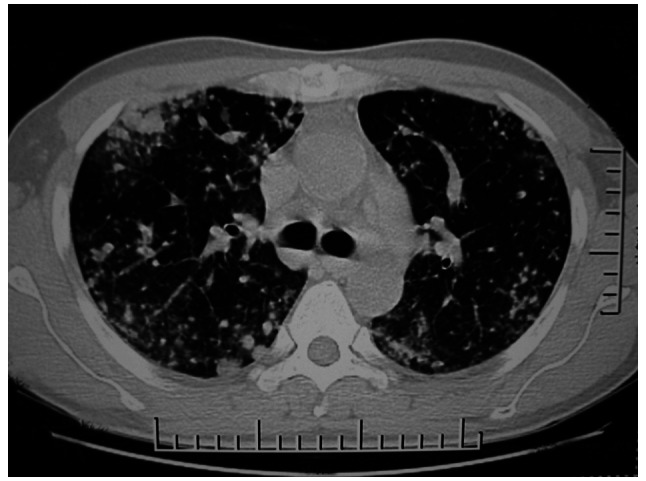
The computed tomography (CT) of the chest shows that the multiple bilateral nodules progressed rapidly following surgery.

PEH disease progressed rapidly in this patient one month after pulmonary surgery. The T1-weighted magnetic resonance imaging (MRI) section examination revealed a nodular lesion in the brain, which was strongly suggestive of brain metastasis (Fig. 4). The CT revealed a spreading of the nodules throughout both lungs three months after surgery (Fig. 5). At this point, the patient began treatment with one cycle of chemotherapy with cisplatin, paclitaxel and endostar (15 mg/day for 14 consecutive days). The patient demonstrated improvements in dyspnea and a dramatic improvement in their clinical status. However, no change in the size of the pulmonary nodules over the period of chemotherapy was observed. The patient subsequently received another two cycles (two, bi-weekly) of chemotherapy treatment with carboplatin, paclitaxel and endostar. No significant reduction was observed in the tumor size and number, and the disease progressed. Following three months of stabilization, progression of the disease was evident. Therefore, the patient was discharged without further treatment. The patient survived for six months following the initial diagnosis.

### Case 2

The patient was a 54-year-old female, non-smoker, who complained of chest pain, dyspnea and a dry cough for 11 months. A chest CT scan revealed intrapulmonary masses in the bilateral superior lobes, and a small right pleural effusion. Abdominal and pelvic CT scans did not reveal any lesions. A thoracoscopic lung biopsy from the right superior lobe was performed in order to examine the nodules. The postoperative course of the patient during follow-up was uneventful. Examination of the nodular sections revealed clusters of neoplastic cells as well as individual tumor cells. The normal pulmonary architecture was replaced by alveoli containing nodules of neoplastic cells and matrix. The histological features of pulmonary epithelioid hemangioendothelioma (EHE) were evident with confirmatory CD31 and CD34 immunohistochemical stains. No markers of mesothelial and muscular differentiation were observed. As a result, the patient was diagnosed with PEH. Immediately following confirmation of the diagnosis, combination chemotherapy with carboplatin, paclitaxel and bevacizumab (15 mg/kg) was initiated for six cycles, without distinct toxicities. The stabilization of the disease was evident, as the chest pain gradually subsided. Following eight months of stabilization, progression of the disease was evident. The patient survived for 15 months following the initial diagnosis.

### Case 3

A 44-year-old female was admitted to our hospital for pleuritic pain and an irritable cough for two months. The patient did not have a history of smoking. A chest CT scan revealed innumerable nodules of various sizes (≤1.5 cm in diameter) in both lungs. Brushing and transbronchial biopsies demonstrated an inflammatory response with numerous eosinophils and no evidence of malignancy. A thoracoscopic lung biopsy from the superior middle lobe was performed. Microscopically, proliferation of histiocytic-like tumor cells with eosinophilic cytoplasm and round nuclei, and without apparent atypical morphology, were observed. Some of the cells contained intracytoplasmic vacuoles. Immunohistologically, the tumor cells were immunoreactive for CD31 and CD34, but negative for cytokeratin, S-100, CD68 and factor VIII. As a result of the examination and the laboratory findings, the patient was diagnosed with PEH. Six cycles of combination chemotherapy of carboplatin and paclitaxel were initiated. The chest CT revealed that there was no significant change in the size of the lung nodules over the period of chemotherapy, and no new metastatic lesions were detected. The patient’s symptoms of pleuritic pain and an irritable cough were improved, and a marked improvement in clinical status was observed. Following 10 months of stabilization, progression of the disease was evident. The patient survived for 25 months following the initial diagnosis.

## Discussion

EHE (formerly known as IVBAT) is an uncommon tumor of vascular endothelial origin, with an intermediate course between hemangioma and conventional angiosarcoma (2–5). The disease was originally described by Dail and Liebow, in 1975, as an IVBAT (1).

Immunohistochemical and electron microscopy studies revealed that IVBAT is of endothelial origin, and the tumor was renamed PEH (4–8). PEH is also of multicentric origin, and extrapulmonary lesions arise from the liver, bone, soft tissue and skin. The 2004 World Health Organization Classification of Tumors regards PEH as a low- to intermediate-grade vascular neoplasm (9).

A previous study reviewed 15 cases of this rare tumor that involved the lungs (10), while another reviewed a total of 93 cases from the literature published in 2006 (11). The authors found that the average age of patients suffering from PEH was 40.1±17.3 years, and 73% of patients were female. Almost half of patients were asymptomatic (49.5%). Reported symptoms were dyspnea and a cough (18.3% each), chest pain (16%), hemoptysis and weight loss (6.5% each). In the present study, two of our patients who were diagnosed with PEH complained of dyspnea and chest pain, one presented with hemoptysis and all patients had a dry cough. Only one patient presented with an indolent course developing over several months prior to the diagnosis.

The hallmark of PEH that is evident on chest radiographs or CT scans is the presence of multiple perivascular nodules with well- or ill-defined margins in both lungs; however, a single cavitary nodule is also observed in PEH patients (12). Radiographically, bilateral multiple nodular opacities are the most common presentation (62–65% in a number of studies) (3,13). All three of our patients demonstrated multiple nodular shadows in both lungs by chest radiography.

PEH is difficult to diagnose and thus the diagnosis may be delayed. There are only two cases of PEH diagnosed by transbronchial biopsy that have been reported in the literature (14,15). The majority of previously reported cases of PHE in the literature were diagnosed using open-lung or thoracoscopic biopsy specimens (14). For this reason, our patients underwent surgery and a thoracoscopic wedge resection of the lung to confirm the diagnosis of PHE.

The diagnosis of PEH is conducted on the basis of histological features and is confirmed immunohistochemically (4,16). Immunohistochemistry for PEH reveals that all tumors display immunoreactivity to some or all of the vascularendothelial markers (CD31, CD34 and factor VIII) (17). The tumors stained positively for CD34 in all three of our patients. Immunostaining for CD31 was positive in two of our patients, while factor VIII was positive in one patient.

The poor prognostic factors of PEH include the presence of respiratory symptoms or pleural effusion revealed by chest radiography on presentation; extensive intravascular, endobronchial or interstitial tumor spreading, hepatic metastases; peripheral lymphadenopathy; the presence of spindle cells in the tumor (4); and, particularly, pleural hemorrhagic effusions and hemoptysis (18).

In patients with pleural effusion or hemoptysis, the median survival is less than one year. Furthermore, Amin *et al*(11) demonstrated that respiratory symptoms and the presence of pleural effusion were independent negative predictors of survival. One of our patients had hemoptysis, which placed him in the poor prognostic group.

There is no established standard treatment for PEH, due to the rarity of the disease. Surgical resection should be performed if possible, and chemotherapy appears to have little effect. Watchful waiting is an acceptable option, particularly in asymptomatic patients (19). Radiotherapy is not effective in certain patients, due to the slow growth of the tumor cells (20–22). Surgical resection is chosen when the PEH lesion is solitary or when the number of lesions is limited. Regular follow-up with no active therapy has been employed in asymptomatic patients with diffuse lesions (2,3). Although its etiology remains unknown, immunohistochemical and electron microscopy studies have revealed that PEH is of endothelial origin. Lymphatic dissemination is extremely rare, thus supporting the endothelial origin of the tumor. Vascular endothelial growth factor (VEGF) and the VEGF receptor were found on EHE tumor cells (23,24), suggesting that VEGF may be involved in the pathogenesis of EHE, and that VEGF inhibitors may be a potential treatment for EHE.

However, no standard therapy has been established for a case of the disease with bilateral multiple nodules (18,25). Certain studies have suggested that certain patients with bilateral multiple nodules may benefit from chemotherapy and immunotherapy. Although there are a small number of case reports of the effectiveness of chemotherapy, a general standard of chemotherapy has been established for PEH (14). We reviewed the literature on those patients who received chemotherapy and immunotherapy (Table I) (2,25–37). Notably, patients with PEH demonstrated a good partial response to chemotherapy with carboplatin, paclitaxel, bevacizumab, thalidomide and α-interferon. Belmont *et al*(30) studied the effect of carboplatin, paclitaxel and bevacizumab in PEH, and found that one patient experienced a clinical benefit. We decided to apply the combination of carboplatin, paclitaxel and endostar as the first-line chemotherapy for the patient in Case 1, while the patient in Case 2 was treated with bevacizumab. For the patient in Case 3, we selected carboplatin and paclitaxel as the chemotherapy. During treatment, all three patients demonstrated stabilization of the disease and an improvement in clinical status. However, no change in the size of the pulmonary nodules over the period of chemotherapy was observed in any of the three patients.

It has been demonstrated that patients with PEH show a good partial response to combination chemotherapy with carboplatin, paclitaxel and bevacizumab (30); this particular study was the first report of the effective use of bevacizumab in treating PEH. Although no tumor response was observed in another case, the second study using bevacizumab to treat PEH, treatment with an antiangiogenic agent was observed to be relatively effective considering that PEH is a tumor of vascular origin (31). In our cases, two patients demonstrated a good partial response to combination chemotherapy with carboplatin, paclitaxel, endostar or bevacizumab. Endostar, a recently introduced recombinant human endostatin, has been considered to be one of the most valuable antiangiogenic agents. This is the first report in which PEH is treated with endostar. Although our treatment regimens failed to stop the progress of this disease, our study may provide insights into treating this rare tumor. Further studies of treatment are warranted to provide a rational basis for using the combination chemotherapy to treat PEH.

In summary, we described three patients with PEH who were treated with combination chemotherapy (carboplatin, paclitaxel, endostar or bevacizumab). All patients demonstrated stabilization of the disease and a dramatic improvement in clinical status. These cases potentially aid efforts to find an effective treatment method for this rare disease. Further research and case reports are required to contribute to the data regarding the clinical findings and the natural history of this rare tumor.

## Figures and Tables

**Table I:** Summary of patients with pulmonary epithelioid hemangioendothelioma treated with chemotherapy and immunotherapy.

First author (Refs.)	No.	Median age at detection (years)	M/F	Pulmonary nodules	Treatment	Response
Kitaichi M (2)	5	Not reported	Not reported	Multiple bilateral	Mitomycin C, 5 fluorouracil, cyclophosphamide, vincristine, tegafur or cisplatin	Progressive disease
Pinet C (25)	1	50	0/1	Bilateral pleural	Carboplatine and etoposide	Complete remission
Erasmus JJ (26)	1	63	0/1	Multiple bilateral	α-interferon	Partial response
Roudier-Pujol C (27)	1		0/1	Multiple bilateral	α-interferon	Partial remission
Marsh K (28)	1	24	0/1	Multiple bilateral	Azathioprine	No deterioration
Ledson MJ (29)	1	24	0/1	Multiple bilateral	Azathioprine	No deterioration
Belmont L (30)	1	41	1/0	Multiple bilateral	Carboplatin, paclitaxel and bevacizumab	Partial remission
Kim YH (31)	1	44	0/1	Multiple bilateral	Carboplatin, paclitaxel and bevacizumab	Progressive disease
André ST (32)	1	65	0/1	Pleural presentation	Carboplatin, etoposide	Progressive disease
Lopes T (33)	1	51	1/0	Multiple bilateral	Carboplatin, etoposide and bevacizumab	Progressive disease
Lee YJ (34)	1	31	0/1	Pleural EHE extending to lung and bone	Adriamycin, dacarbazine and ifosfamide	Stabilized during chemotherapy
Radzikowska E (35)	1	62	0/1	Multiple bilateral	α-interferon	Partial remission and then stabilization of the disease
Endo T (36)	1	69	1/0	Lung, pleura, ribs extending to brain	α-interferon	Progressive disease
Marsh RW (37)	1	57	0/1	Multiple bilateral	Methotrexate and α-interferon	Progressive disease (methotrexate); partial remission and then stabilization of the disease (α-interferon)

M, male; F, female; EHE, epithelioid hemangioendothelioma.
